# Benefits of the Non-Steroidal Mineralocorticoid Receptor Antagonist Finerenone in Metabolic Syndrome-Related Heart Failure with Preserved Ejection Fraction

**DOI:** 10.3390/ijms24032536

**Published:** 2023-01-28

**Authors:** Ixchel Lima-Posada, Yohan Stephan, Matthieu Soulié, Roberto Palacios-Ramirez, Benjamin Bonnard, Lionel Nicol, Peter Kolkhof, Frederic Jaisser, Paul Mulder

**Affiliations:** 1 Centre de Recherche des Cordeliers, UMRS 1138, INSERM, Sorbonne Université, Université Paris Cité, 75006 Paris, France; 2 INSERM EnVI UMR 1096, Univ Rouen Normandie, 76183 Rouen, France; 3 Cardiovascular Precision Medicines, Research and Early Development, Pharmaceuticals, Bayer AG, 42113 Wuppertal, Germany; 4 INSERM, Clinical Investigation Centre 1433, French-Clinical Research Infrastructure Network (F-CRIN) INI-CRCT (Cardiovascular and Renal Clinical Trialists), 54500 Nancy, France

**Keywords:** diabetes, heart failure, mineralocorticoid receptor antagonist, diastolic dysfunction, finerenone

## Abstract

The mineralocorticoid receptor (MR) plays an important role in the development of chronic kidney disease (CKD) and associated cardiovascular complications. Antagonizing the overactivation of the MR with MR antagonists (MRA) is a therapeutic option, but their use in patients with CKD is limited due to the associated risk of hyperkalemia. Finerenone is a non-steroidal MRA associated with an improved benefit-risk profile in comparison to steroidal MRAs. In this study, we decided to test whether finerenone improves renal and cardiac function in male hypertensive and diabetic ZSF1 rats as an established preclinical HFpEF model. Finerenone was administered at 10 mg/kg/day for 12 weeks. Cardiac function/hemodynamics were assessed in vivo. ZSF1 rats showed classical signs of CKD with increased BUN, UACR, hypertrophy, and fibrosis of the kidney together with characteristic signs of HFpEF including cardiac fibrosis, diastolic dysfunction, and decreased cardiac perfusion. Finerenone treatment did not impact kidney function but reduced renal hypertrophy and cardiac fibrosis. Interestingly, finerenone ameliorated diastolic dysfunction and cardiac perfusion in ZSF1 rats. In summary, we show for the first time that non-steroidal MR antagonism by finerenone attenuates cardiac diastolic dysfunction and improves cardiac perfusion in a preclinical HFpEF model. These cardiac benefits were found to be largely independent of renal benefits.

## 1. Introduction

The The prevalence of diabetes has increased continuously in the past years, representing a major challenge for the society and public health systems around the world [1]. Chronic kidney disease (CKD) and heart failure are the principal complications of diabetes [2,3]. Diabetes and other cardiometabolic risk factors contribute to de development of HFpEF pathogenesis through endothelial dysfunction, inflammation, and oxidative stress [4,5,6]. Diabetic patients with impaired renal function are at higher risk to suffer from cardiovascular events than kidney failure, therefore, it is important to have a global approach to limit CKD progression and cardiovascular disease (CVD) in diabetic patients in order to reduce the incidence of cardiovascular morbidity and mortality. 

The mineralocorticoid receptor (MR) plays an important role in the development of CKD and associated cardiovascular complications especially in case of type 2 diabetes. Antagonizing the overactivation of the MR is a therapeutic strategy largely studied in cardiovascular [7,8,9,10] and kidney diseases models [11,12,13,14], showing the benefit of using mineralocorticoid receptor antagonists (MRA) to delay the progression of the disease. However the use of the available steroidal MRA is limited in target patient populations with compromised renal function due to the risk of hyperkalemia [15].

Finerenone is a non-steroidal mineralocorticoid receptor antagonist with different and specific properties compared to classical steroidal MRAs such as spironolactone and eplerenone [16]. Finerenone has a protective action in preclinical models of kidney and cardiovascular diseases [17,18,19]. Finerenone has been associated with a lower risk of developing hyperkalemia compared with spironolactone in the ARTS phase 2a trial [20]. Two large clinical trials have been recently published showing the beneficial effects of finerenone in stage 1 to 4 CKD patients with type 2 diabetes (T2D) on top of optimal renin-angiotensin-system (RAS) therapy including angiotensin converting enzyme inhibitors (ACEi) or angiotensin receptor blockers (ARB): the FIDELIO-DKD trial demonstrated the beneficial effect of finerenone compared with placebo, lowering the risks of CKD progression and cardiovascular events in 5734 patients with advanced CKD [21] and the FIGARO-DKD trial that included 7437 patients with type 2 diabetes and less advanced CKD showed improved cardiovascular outcomes compared with placebo [22]. Of note the incidence of hyperkalemia-related discontinuation (2.3% and 0.9%, in the finerenone and placebo group, respectively) was markedly lower than any dual RAS blockade in previous trials in CKD patients with T2D [23] or with spironolactone on top of RAS blockade in CKD [24].

Our group has previously shown that finerenone prevented from systolic and diastolic dysfunctions and changes in the heart structure associated with non-diabetic CKD induced by 5/6 nephrectomy in mice [25]. We also reported that finerenone opposed metabolic syndrome-related diastolic cardiac dysfunction and nephropathy in the Zucker rat, a model with insulin resistance and diabetic disease [17]. The aim of this study was to test whether finerenone improves renal and/or cardiac functions in the ZSF1 rat, a model with metabolic syndrome-related heart failure with preserved ejection fraction (HFpEF) which has repeatedly been reported to show distinct features of HFpEF, such as an increased left ventricular (LV) end diastolic pressure, LV hypertrophy, diastolic dysfunction, lung congestion and left atria remodeling, while maintaining a preserved ejection fraction (EF) [26].

## 2. Results

### 2.1. Renal and Cardiac Characteristics in the ZSF-1 Rats

The obese ZSF1 rats develop a model of diabetic nephropathy associated with cardiac dysfunction.

ZSF1 and lean control rats were followed for 12 weeks, and renal and cardiac damages were analyzed.

**Figure 1:**
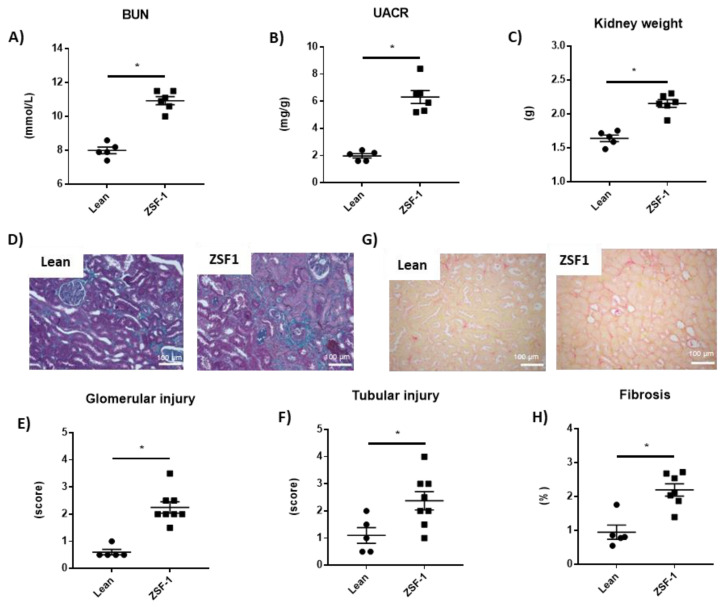
Renal injury developed in obese ZSF-1 rats after 12 weeks. Blood urea nitrogen levels (**A**), urine albumin to creatinine ratio (**B**), and kidney weight (**C**). Representative light microphotographs (20× magnification) of rat kidney sections stained with Masson Trichrome (**D**) and Sirius red (**G**); glomerular (**E**) and tubular injury quantification (**F**), kidney fibrosis (**H**). Student’s *t*-test was used for statical analysis, *n* = 5–8. * *p* < 0.05 vs. lean.

We observed increased blood urea nitrogen levels (BUN) (Figure 1A), urine albumin-creatinine ratio (UACR) (Figure 1B), renal hypertrophy (Figure 1C), glomerular (Figure 1D–E) or tubular injuries (Figure 1D,F) and interstitial kidney fibrosis (Figure 1G,H) in the ZSF1 rats compared to the control lean rats.

**Figure 2:**
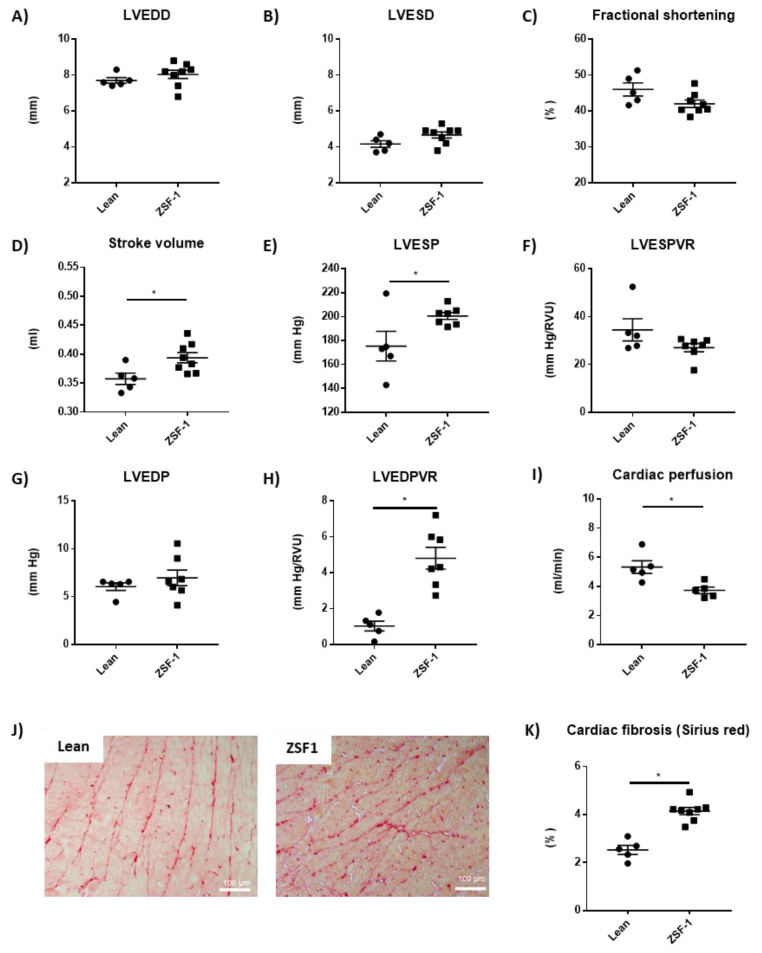
Impaired LV diastolic function is associated with a decrease in left ventricle perfusion and myocardial interstitial fibrosis in the ZSF1 rats. Left ventricle end-diastolic diameter (**A**), left ventricle end-systolic diameter (**B**), fractional shortening (**C**), stroke volume (**D**), left ventricle end-systolic pressure (**E**), left ventricle end-systolic pressure–volume relationship (**F**), left ventricle end-diastolic pressure (**G**), left ventricle end-diastolic pressure–volume relationship (**H**) and left ventricle tissue perfusion (**I**). Representative light microphotographs (20× magnification) of rat kidney sections stained with Sirius red (**J**) and kidney fibrosis quantification (**K**). Student’s *t*-test was used for statical analysis, *n* = 5–8. * *p* < 0.05 vs. lean.

12 weeks after the beginning of the experimental period, echocardiography showed that, compared to lean control rats, ZSF1 rats have similar LV end-diastolic diameter (Figure 2A), a trend to increase LV end-systolic diameter (Figure 2B), together with reduced fractional shortening (*p* = 0.06 vs. lean) (Figure 2C) and increased stroke volume (Figure 2D) whilst cardiac output was similar (data not shown). At the end of this 12 weeks experimental period, invasive LV hemodynamic studies were performed. Systolic blood pressure was strongly increased in ZSF1 rats compared to lean control rats (mmHg: 175 ± 13.2 vs. 204 ± 1.5, *n* = 5–7, mean +/− SEM, *p* = 0.026). LV pressure–volume curves showed an increase in the LV end-systolic pressure (LVESP) in the ZSF1 rats compared to lean (Figure 2E) and similar LV end-systolic pressure–volume relationship (LVESPR) (Figure 2F); LV end-diastolic pressure (LVEDP) were similar between groups (Figure 2G) whilst LV end-diastolic pressure–volume relationship (LVEDPVR) is increased in ZSF1 rats compared to lean (Figure 2H). No changes were observed in dP/dtmax, dP/dtmin, and Tau (Supplementary Table S1). These results suggested impaired LV contractility and compliance in ZSF1 compared to lean which are the early signs of diastolic dysfunction. Myocardial perfusion, which takes place during the diastolic phase in the cardiac cycle, was assessed by magnetic resonance imaging (MRI) and showed a decrease in the perfusion of the left ventricle in the ZSF1 rats compared to lean control rats (Figure 2I). The worsening in the LV diastolic function was associated with myocardial interstitial fibrosis in the ZSF1 rats compared to the control rats (Figure 2J–K). Together these results show that LV diastolic dysfunction was associated with the increase in LVESP and LVEDPVR. We also found a reduction in cardiac perfusion that could be responsible for the increase in oxidative stress and contribute to the development of fibrosis, influencing factors of the diastolic dysfunction we see in the ZSF1 rats.

### 2.2. Impact of Finerenone in Renal and Cardiac Dysfunction in the ZSF-1 Rats

**Figure 3:**
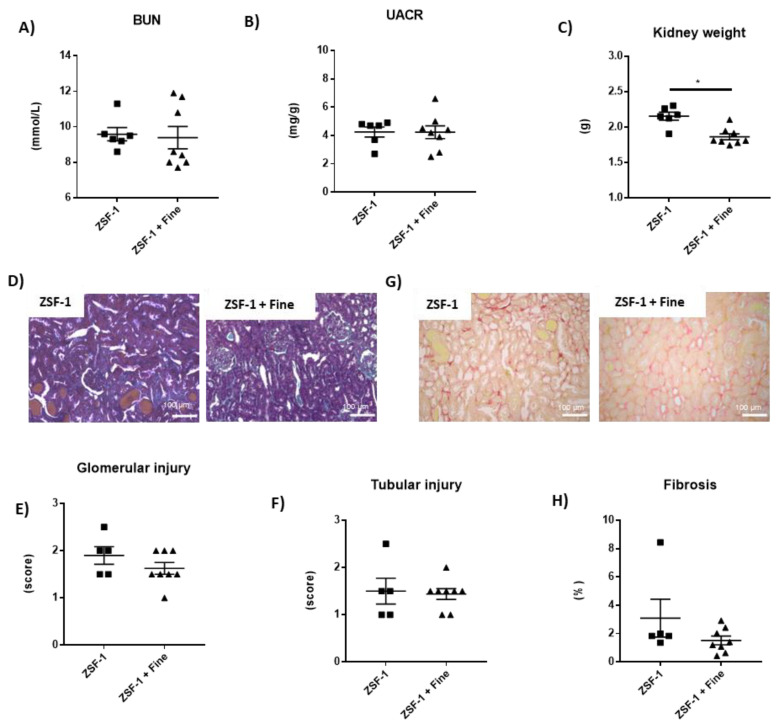
Kidney hypertrophy is reduced by finerenone treatment after 12 weeks. Blood urea nitrogen levels (**A**), urine albumin to creatinine ratio (**B**), and kidney weight (**C**). Representative light microphotographs (20× magnification) of rat kidney sections stained with Masson Trichrome (**D**) and Sirius red (**G**); glomerular (**E**) and tubular injury quantification (**F**), kidney fibrosis (**H**). Student’s *t*-test was used for statical analysis, *n* = 5–8. * *p* < 0.05 vs. ZSF-1.

We next studied the impact of the non-steroidal MRA finerenone on renal and cardiac parameters in the ZSF1 diabetic model. The treatment of finerenone for 12 weeks had no impact on BUN (Figure 3A), UACR (Figure 3B), glomerular and tubular injuries (Figure 3D–F) or renal fibrosis (Figure 3G,H), whilst it decreased the kidney hypertrophy (Figure 3C), compared to the non-treated animals. Finerenone has no impact on plasma potassium content (mmol/L: 3.8 ± 0.2 vs. 3.8 ± 0.2, *n* = 6–8, mean +/− SEM, *p* = ns).

**Figure 4:**
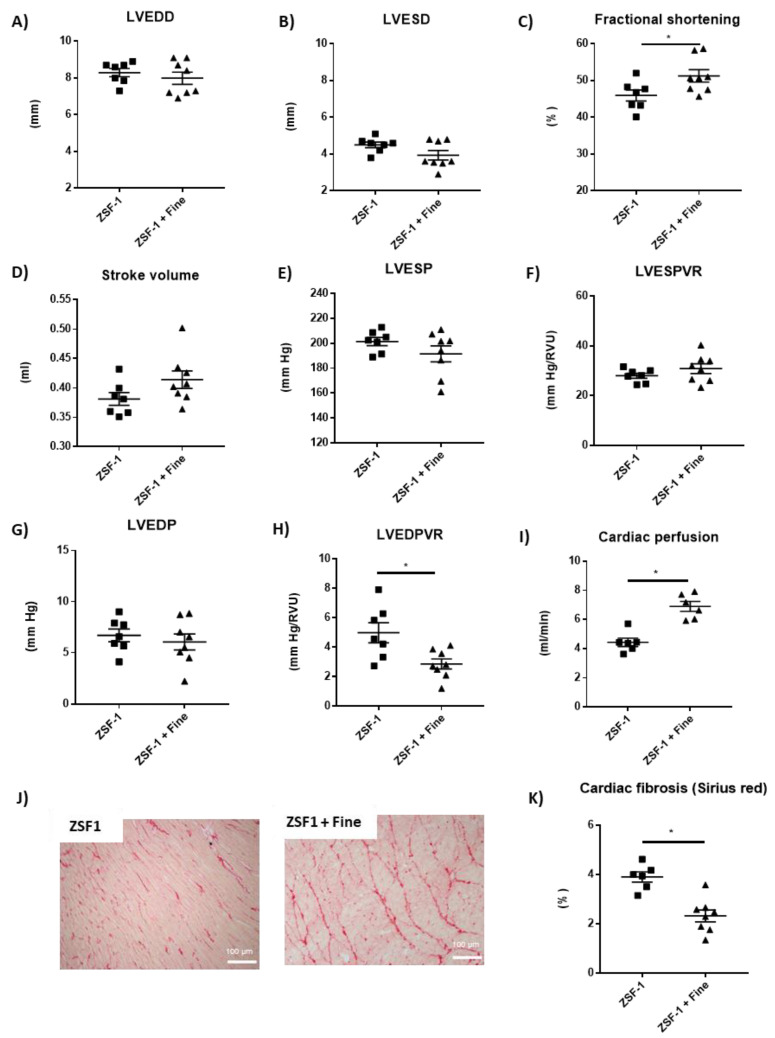
Finerenone ameliorates diastolic dysfunction and fibrosis together with better myocardial perfusion. Left ventricle end-diastolic diameter (**A**), left ventricle end-systolic diameter (**B**), fractional shortening (**C**), stroke volume (**D**), left ventricle end-systolic pressure (**E**), left ventricle end-systolic pressure–volume relationship (**F**), left ventricle end-diastolic pressure (**G**), left ventricle end-diastolic pressure–volume relationship (**H**) and left ventricle tissue perfusion (**I**). Representative light microphotographs (20× magnification) of rat kidney sections stained with Sirius red (**J**) and kidney fibrosis quantification (**K**). Student’s *t*-test was used for statical analysis, *n* = 5–8. * *p* < 0.05 vs. ZSF-1.

Finerenone treatment did not alter LV end-diastolic diameter (Figure 4A) and LV end-systolic diameter (Figure 4B) but increased fractional shortening (Figure 4C), whilst stroke volume (Figure 4D) and cardiac output (data not shown) were not modified. Systolic blood pressure was not modified by finerenone (mmHg, 204.6 ± 3.1 vs. 195.4 ± 5.5, *n* = 8, mean +/− SEM, *p* = ns). LV hemodynamic studies showed similar LV end-systolic pressure (Figure 4E), LV end-systolic pressure–volume relationship (Figure 4F), and LV end-diastolic pressure (Figure 4G) between groups, whilst LV end-diastolic pressure–volume relationship was reduced with finerenone treatment compared to non-treated animals (Figure 4H), suggesting amelioration of the diastolic dysfunction observed in the ZSF1 rats. Although not statistically significant (7% increase in dP/dtmin and 11% reduction in Tau induced by finerenone when compared to untreated ZSF-1) all the parameters suggest an improvement of LV diastolic function while all systolic parameters are not modified by finerenone (Supplementary Table S1). Left ventricle myocardial perfusion was strongly improved in the finerenone-treated group compared to the non-treated ZSF1 rats (Figure 4I). The improvement in the LV diastolic function in the finerenone-treated rats was associated with less myocardial interstitial fibrosis compared to non-treated animals (Figure 4J–K).

### 2.3. Involvement of Nitric Oxide (NO) to the Effects of Finerenone on LV Function and LV Tissue Perfusion

We next studied the effect of nitric oxide synthase (NOS) inhibition on the beneficial cardiac effect of finerenone. After 7 days of *N*(ω)-nitro-l-arginine methyl ester (l-NAME) treatment, we observed a significant reduction in the heart rate, without affecting systolic blood pressure. Interestingly, l-NAME depleted the beneficial effects of finerenone on the indices of LV diastolic function as seen in LVEDP, Tau, dP/dtmin, and LVEDPVR, but did not modify the indices of LV systolic function (Table 1). Lastly, L-NAME blunted the increase in LV tissue perfusion induced by finerenone treatment (Table 1).

## 3. Discussion

Together these results show that finerenone treatment reduced kidney hypertrophy and cardiac fibrosis and improved cardiac diastolic function and perfusion in diabetic ZSF1 rats. This is to the best of our knowledge the first published report demonstrating a benefit of MR antagonism in this particular metabolic syndrome-associated preclinical HFpEF model. 

The obese ZSF1 rats is a model of diabetic nephropathy associated to cardiac dysfunction. The ZSF1 rats that has been previously characterized as a model of HFpEF. Several studies indeed reported the cardiac characteristics of the model, with increased LV mass, LVEDP, LVESPVR, LVESP, LVEDD, LVEDV (left ventricular end-diastolic volume), E/E’, E/A; decreased HR, cardiac index, RVEF (right ventricle ejection fraction), effort resistance, LAEF (left atrial ejection fraction); no changes in LVEF (left ventricle ejection fraction), cardiac output and Tau [26,27,28,29,30,31].

The role of MR overactivation mainly assessed using MR antagonists has been confirmed in various diabetic animal models, reporting the benefit of MR blockade on renal injury [32,33,34,35] and cardiac dysfunction [36,37,38,39,40,41]. The lack of beneficial effect of finerenone in renal characteristics in our model is in contrast with other findings in different rodent diabetic models [34,35,42]. These differences may rely to the type of model used (such as type 1 diabetes in rat [32,33,34,35,43] or type 2 diabetes in *db/db* mice [43,44,45]. In these reports, MRA treatment slow down albuminuria independently of the type of MRA (steroidal or non-steroidal). The MRA Eplerenone used in our previous study has no effect at all in the ZSF1 rat model in absence or in presence of AngII co-treatment [46]. The absence of albuminuria lowering effect of the MRA finerenone in ZSF1 rats may be intrinsic to the model (or to the treatment duration), but other studies are needed to clarify this point. The underlying mechanisms of MR blockade mainly involve a reduction of organ fibrosis and inflammation but a direct effect on adipose tissue and its metabolic consequences [47] may indirectly have beneficial impact on cardiorenal functions. A reduction of local oxidative stress has also been proposed. While most of the pharmacological studies used classical steroidal MRAs like spironolactone [38,39,41] or eplerenone [34,35,40], only two studies assessed the impact of the non-steroidal MRA finerenone in this context. Lachaux et al. reported that finerenone blunted metabolic syndrome-related diastolic cardiac dysfunction and nephropathy in the obese Zucker rat model [17]. Using a complex model associating high salt intake/uninephrectomy in the diabetic *db/db* mice, Hirohama et al. reported that both hypertension and renal injury were ameliorated by finerenone [44].

The activation of the MR in diabetes may result from a combination of various mechanisms: increased ligand levels, either aldosterone for which increased synthesis has been reported from the adrenal upon obesity [48] or from local production for example by adipocytes [49], while increased MR activation by glucocorticoid may also occur, especially in cells where HSD2 is not expressed, together with the induction of local HSD1 activity associated to diabetes [50]. Increased MR expression has been reported in podocytes [51] and this may also occur in other cell types [52] leading to global overactivation of MR-dependent pathways. Of note, ligand independent activation of the MR by oxidative stress has been proposed and showed to be at least partly mediated by Rac1 as a target for the beneficial renal effects of finerenone in the the high-salt diet/uninephrectomy/diabetic mouse model (HS/UNx/db/db) [44].

Myocardial perfusion is critical for the supply of oxygen and nutrients, deliveries of hormones and growth factors or vasoactive agents to improve or maintain adequate coronary perfusion for myocardium contraction efficacy and maximal exercise capacity, especially in HFpEF [53]. Under physiological conditions coronary arterial perfusion mainly occurs during relaxation and impaired diastolic function reduces myocardial perfusion [54]. The benefit of finerenone on myocardial perfusion we report in the ZSF1 rats is therefore very important in the context of metabolic associated HFpEF and may participate to the global benefit of finerenone on CV outcomes noticed in clinical trials. Down-regulation of endothelial nitric oxide synthase (eNOS) expression and activation has been reported in ZSF1 model in the kidney and heart [55,56] and previous studies showed that that finerenone regulates the production of NO through the regulation of eNOS in heart tissue [9,17,57]. We therefore studied the effect of Nitric oxide synthase (NOS) inhibition on the beneficial cardiac effect of finerenone. One underlying mechanism relies in part to an increase NO availability as we showed that L-NAME perfusion, preventing NO production, indeed blunted the benefit of finerenone on myocardial perfusion. 

The clinical impact of antagonizing the MR in diabetic patients has been highlighted recently in two major clinical trials, involving more than 13,000 patients. The FIDELIO-DKD and the FIGARO-DKD showed that finerenone improved renal and cardiovascular outcomes compared with placebo [21,22]. The present preclinical study suggests that the cardiovascular benefits are, at least in part, related to the improvement of diastolic function, decreased interstitial cardiac fibrosis and improved myocardial perfusion. Accordingly, a phase III study called FINEARTS-HF (NCT04435626) to evaluate the efficacy and safety of finerenone on morbidity and mortality in patients with HF and a LVEF of ≥40% is currently ongoing. The study plans to enroll 6000 patients suffering from HF with mid-range and preserved ejection fraction (HFmrEF and HFpEF). FINEARTS-HF is currently the largest outcome trial investigating an MRA in HFpEF (Last Update Posted at ClinicalTrials.gov: 13 September 2022).

An important question is whether combining MRAs, especially finerenone, with with Sodium/glucose cotransporter-2 inhibitors (SGLT2i) would add a benefit in comparison to the use of each antagonist separately in diabetic kidney disease (DKD) patients on the renal and CV outcomes. Indeed the selective SGLT2 inhibitor empagliflozin improved systolic blood pressure and reduced the left ventricle weight and volume as well as posterior wall thickness in ZSF1 rats but did not improve left ventricle filling estimated by the E/E′ ratio in echocardiographic study [58]. Empagliflozin also normalized NO-mediated endothelium-dependent relaxation in ZSF1 rats [58]. Chronic treatment with the dual SGLT-1-2 inhibitor sotagliflozin was effective in mitigating left atria cardiomyopathy in this rat model of metabolic syndrome-related HFpEF [26]. Since the benefits of MRAs only partly overlap with those of SGLT2i, an additive benefit (or even a synergistic action) may be expected. Such a combination of finerenone and empagliflozin confer renal and CV protection in a preclinical mouse model of hypertension-induced cardiorenal disease [59]. Combining the SGLT2 inhibitor dapagliflozin with the steroidal MRA eplerenone resulted in a robust additive UACR-lowering effect in the randomized cross-over clinical trial ROTATE-3 but long-term impact on renal or CV outcomes was not assessed [60]. The Phase II CONFIDENCE study (NCT05254002) will investigate finerenone plus empagliflozin compared with either finerenone or empagliflozin alone in about 870 patients with CKD and T2D.

## 4. Materials and Methods

The data generated during and/or analyzed during the current study are available from the corresponding author upon reasonable request.

### 4.1. Experimental Design

**Figure 5:**
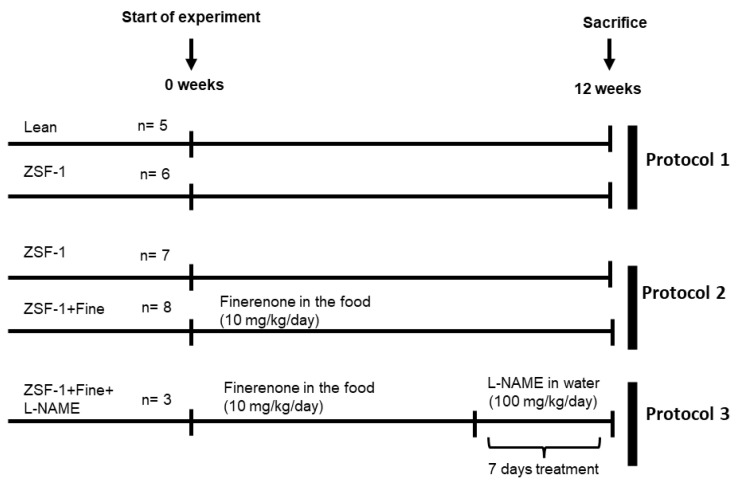
Scheme of the experimental protocol. Fine, finerenone; l-NAME, *N*(ω)-nitro-l-arginine methyl ester.

Experiments were approved by the Darwin ethics committee of Sorbonne University (#18317-2019010411586046) approved on 15 July 2019, and conducted according to the INSERM animal care and use committee guidelines. 26 male 12-weeks-old ZSF1 rats (obese and lean control) were purchased from Charles River and 3 separate protocols were performed (Figure 5). Protocol 1: ZSF1 lean (control *n* = 5) and ZSF1 (*n* = 6) were included to characterize the model. Protocol 2: to evaluate the impact of finerenone treatment in ZSF1 rats, ZSF1 without treatment (*n* = 7) and ZSF1 treated (*n* = 8) with finerenone in the food (10 mg/kg/day) were included. Protocol 3: to test the effect of NOS inhibition in ZSF-1 rats, ZSF1 rats treated with finerenone were also treated with l-NAME (Sigma Aldrich. Saint-Louis, MO, USA) for 7 days at the end of the experimental period (100 mg/kg/day). The animals were followed for 12 weeks and housed in a climate-controlled facility with a 12-h light/12-h dark cycle and provided free access to water and food. The welfare of the rats was monitored throughout the study. Physiological analyses were performed between 11–12 weeks of follow-up. Animals were sacrificed at 12 weeks of study. Tissues were collected, weighted, and rinsed in ice-cold Dulbecco’s phosphate buffered saline (DPBS; ThermoFisher, Waltham, MA, USA); the kidney and heart were cut in two parts, one for histology and the other for molecular analysis that was frozen in liquid nitrogen and stored at −80 °C.

### 4.2. Biochemical Studies

24-hr urine collection was performed using metabolic cages in all studied groups 12 weeks after the beginning of the experiment. We determined plasma urea and potassium; and urinary albumin and creatinine levels with an automatic analyzer (Catalyst one, IDEXX. Westbrook, ME, USA). UACR was calculated as urinary albumin (mg)/urinary creatinine (g).

### 4.3. Echocardiography

Transthoracic Doppler echocardiographic studies were performed after 11 weeks of treatment in anesthetized rats with isoflurane (ISO-VET^®^, 3% for induction, 2% for maintenance) using an echocardiographic system (VIVID 7, GE) equipped with an 8–5 MHz transducer. Briefly, a two-dimensional short-axis view of the left ventricle was obtained at the level of the papillary muscle to record M-mode tracings. Left ventricular diameters were measured following the American Society of Echocardiology leading-edge method from at least 3 consecutive cardiac cycles. Left ventricular outflow velocity was measured by pulsed-wave Doppler, and cardiac output was calculated as CO = aortic VTI ● [π ● (left ventricular outflow diameter/2)2] ● heart rate, where VTI is a velocity-time integral [61].

### 4.4. Magnetic Resonance Imaging (MRI)

Left ventricular tissue perfusion was evaluated in anesthetized animals (Brietal™, 50 mg/kg IP) at 11 weeks of treatment, using magnetic resonance imaging (Bruker Biospec 4.7 Tesla, Billerica, Masachusetts, USA) with arterial spin labeling technique. Perfusion images were analyzed with ParaVision 5.0 software (Bruker, Billerica, MA, USA) [62].

### 4.5. Hemodynamic Studies

Hemodynamic studies were performed in the left ventricle. LV hemodynamics was determined by measuring the LV pressure–volume curves after the treatment period. The right carotid artery was cannulated with a micromanometer tipped catheter (SPR 838, Millar Instruments, Houston, TX, USA) in anesthetized rats (Brietal™; 50 mg·kg^−1^, IP). Arterial blood pressure and heart rate were recorded, and after that, the catheter was introduced into the LV for LV pressure recording. We gently occluded the abdominal aorta with a cotton swab to obtain LV pressure–volume loops at baseline and during loading. Data were stored and analyzed by using Millar conductance data acquisition and analysis software (IOX™, EMKA, Velbert, Germany). Finally, we measured or calculated left ventricular end-systolic and end-diastolic pressures, dP/dtmax/min, left ventricular relaxation constant Tau, and slopes of left ventricular end-systolic as well as end-diastolic pressure–volume relations.

### 4.6. Histology

Kidney sections and the left ventricle were collected and immersed in paraformaldehyde fixative solution (Sigma-Aldrich^®^. Burlington, MA, USA). After fixation, the sections were dehydrated and embedded in paraffin. From these sections, 5-μm thick histologic slices were obtained and were stained with Sirius Red for collagen determination in the heart and kidney, and Masson Trichrome staining for kidney structure. For the measurement of kidney fibrosis and structure, slides were examined and 10 microphotographs per sample were obtained under a microscope (Zeiss, Oberkochen, Germany) at 20× magnification. Injured tubules and glomeruli were counted using ZEN 3.1–Zeiss software (Zeiss, Oberkochen, Germany). A semi-quantitative score was graded from 0 to 4. Renal and LV fibrosis was calculated as a percentage of collagen area to the total area of the image of the staining. All analyses were blinded.

### 4.7. Statistical Analysis

The results are presented as the mean  ±  SE. Differences in the means between the two groups for non-repeated variables were compared by Student’s *t*-test. All comparisons passed the normality test (Shapiro–Wilk normality test, in GraphPad Prism 7.04). Analysis was performed using GraphPad Prism 7.4. Results were considered significant when *p* < 0.05.

## 5. Conclusions

In summary, we show for the first time that the non-steroidal MR antagonist finerenone attenuates cardiac diastolic dysfunction and improves cardiac perfusion in a preclinical HFpEF model. These cardiac benefits were found to be largely independent of renal benefits. The ongoing clinical FINEARTS-HF study will analyze the impact of finerenone on CV outcomes in patients with HFmrEF and HFpEF.

## Figures and Tables

**Table 1:** L-NAME impact on cardiac function in ZSF-1 rats treated with finerenone.

	ZSF1 + Fine *n* = 8			ZSF1 + Fine + L-NAME *n* = 3		
SBP	195	±	5.5	202	±	5.8
HR	327.3	±	6.8	291	±	7.0 *
LVESP	191.5	±	6.4	205.4	±	5.5
dP/dt max	11,467.8	±	504.4	9757	±	419.0 *
LVESPVR	30.8	±	1.9	29.7	±	1.5
LVEDP	6.05	±	0.7	14.6	±	3.0 *
dP/dt min	9979.5	±	641.4	4420.6	±	177.4 *
Tau	12.38	±	0.5	17.29	±	0.5 *
LVEDPVR	2.86	±	0.3	3.76	±	0.1 *
LV tissue perfusion	6.9	±	0.3	3.6	±	0.2 *

Data are presented as the mean ± SEM; *n* = 3–8. * *p* < 0.05 vs. ZSF-1 + Fine. (SBP) systolic blood pressure (mmHg), (HR) heart rate (bpm), (LVESP) left ventricle end-systolic pressure (mmHg), (dP/dt max) contractility (mmHg/s), (LVESPVR) left ventricle end-systolic pressure–volume relationship (mmHg/RVU), (LVEDP) left ventricle end-diastolic pressure (mmHg), (dP/dt min) relaxation (mmHg/s), (Tau) time constant of relaxation (msec), (LVEDPVR) left ventricle end-diastolic pressure–volume relationship (mmHg/RVU) and (LV tissue perfusion) left ventricle tissue perfusion (mL/min).

## Data Availability

The data generated during and/or analyzed during the current study are available from the corresponding author upon reasonable request.

## Supplementary Materials

The following supporting information can be downloaded at: https://www.mdpi.com/article/10.3390/ijms24032536/s1.
