# Supplementary material for: Benefits of the Non-Steroidal Mineralocorticoid Receptor Antagonist Finerenone in Metabolic Syndrome-Related Heart Failure with Preserved Ejection Fraction
Source: Int J Mol Sci. 2023 Jan 28;24(3):2536. doi: 10.3390/ijms24032536 (PMC9916671; doi:10.3390/ijms24032536)
Supplement: Supplementary file 1 [file ijms-24-02536-s001.zip › ijms-2134875-supplementary.pdf]

	Protocol 1		Protocol 2		Protocol 3
	**Lean** n=5	**ZSF1** n=7	**ZSF1** n=7	**ZSF1+fine** n=8	**ZSF1+fine+L-NAME** n=3
**SBP (mm Hg)**	175 ± 13	204 ± 3[&]	203 ± 3	195 ± 6	203 ± 6
**HR (Beats/min)**	316 ± 6	337 ± 7	326 ± 13	327 ± 7	291 ± 7*+
**LVESP (mm Hg)**	175 ± 12	201 ± 3[&]	201 ± 3	192 ± 6	205 ± 6
**dP/dt_{max} (mm Hg/sec)**	9360 ± 492	11750 ± 495[&]	11328 ± 622	11468 ± 504	9757 ± 419*
**LVESPVR (RVU/mm Hg)**	34.5 ± 4.7	27.0 ± 1.7	28.1 ± 1.1	30.8 ± 1.9	29.7 ± 1.5
**LVEDP**	6.04 ± 0.40	6.96 ± 0.81	6.6 ± 7.0	6.05 ± 0.78	14.68 ± 1.96*+
**dP/dt_{min} (mm Hg/sec)**	10920 ± 194	9934 ± 352	9307 ± 442	9980 ± 641	4421 ± 177*+
**Tau (msec)**	10.95 ± 0.14	13.34 ± 0.59[&]	13.83 ± 0.4	12.38 ± 0.47	17.29 ± 0.54*+
**LVEDPVR (RVU/mm Hg)**	1.02 ± 0.27	4.8 ± 0.54[&]	4.98 ± 0.60	2.86 ± 0.33+	3.76 ± 0.13*
**LV tissue perfusion (ml/min 100 g of LV tissue)**	5.33 ± 0.44	3.73 ± 0.24[&]	4.41 ± 0.22	6.96 ± 0.39+	3.61 ± 0.23*

Supplemental Table S1.** **Cardiac function in lean, ZSF-1, ZSF-1+Fine and ZSF-1+Fine +L-NAME rats. *Data are presented as the mean ±SEM; n= 3-8. [&]p<0.05 vs Lean; *p< 0.05 vs ZSF-1 + Fine; [+]p<0.05 vs ZSF1; (SBP) systolic blood pressure (mmHg), (HR) heart rate (bpm), (LVESP) left ventricle end-systolic pressure (mmHg), (dP/dt max) contractility (mmHg/s), (LVESPVR) left ventricle end-systolic pressure volume relationship (RVU/mmHg), (LVEDP) left ventricle end-diastolic pressure (mmHg), (dP/dt min) relaxation (mmHg/s), (Tau) time constant of relaxation (msec), (LVEDPVR) left ventricle end-diastolic pressure volume relationship (RVU/mmHg) and (LV tissue perfusion) left ventricle tissue* [illegible]
